# Verification of the Canadian Laboratory Initiative on Paediatric Reference Intervals (CALIPER) reference values in Croatian children and adolescents

**DOI:** 10.11613/BM.2020.020710

**Published:** 2020-06-15

**Authors:** Renata Zrinski Topić, Jasna Leniček Krleža

**Affiliations:** Department of Laboratory Diagnostics, Children’s Hospital Zagreb, Zagreb, Croatia

**Keywords:** hormone, immunoassay, paediatric, reference value

## Abstract

**Introduction:**

The aim of this study was to examine whether the Canadian Laboratory Initiative on Paediatric Reference Intervals (CALIPER) could be applied to Croatian children and adolescents.

**Materials and methods:**

A total of 295 outpatient healthy children and adolescents of age 1 to 18 were selected using the direct *a posteriori* sampling method. According to current guidelines, 20 samples were tested for each of a total of 51 reference intervals for ferritin, cortisol, dehydroepiandrosterone sulfate, follicle stimulating hormone, lutein stimulating hormone, prolactin, progesterone, sex hormone binding globulin, thyroid stimulating hormone, total testosterone, total thyroxine and total triiodothyronine. Serum samples were analysed on the Beckman Coulter DxI600 immunoassay analyser by chemiluminescence immunoassay method. A reference interval was adopted if < 10% of the results fall outside CALIPER reference interval range. For analytes in which this criterion is not met in the first set of samples, a new set of 20 samples were collected.

**Results:**

After the first set of measurements, 96% of all tested reference intervals were adopted for use. The additional sets of 20 reference subjects were tested for only two reference intervals; follicle stimulating hormone for female aged 1 to 9 years, and irrespective of the gender, sex hormone binding globulin for children aged 8 to 11 years. All results of additional samples were within the specified interval limits.

**Conclusions:**

CALIPER reference intervals for ferritin and 11 hormones defined for Beckman Coulter DxI600 immunoassay analyser can be implemented into the Croatian laboratories and clinical practice.

## Introduction

The concentration of biomarkers in blood and body fluids of a healthy person may vary with age, gender, nutrition, body weight, hormonal status, as well as the analytical method and/or analyser for biomarkers concentration measurement. Therefore, it is necessary to establish reference intervals of diagnostic laboratory tests on referent, or „healthy“, subjects. Reference values based evaluation is one of the options for accurate interpretation of laboratory test results and clinical differentiation between healthy and diseased individuals (*1*).

According to the European Directive on *in vitro* diagnostic medical devices, diagnostic test manufacturers are obliged to provide the information about reference intervals for routine test to their customers (*2*). Unfortunately, paediatric specific reference intervals for neonatal population, children and adolescents are very often not declared in diagnostics test assay sheets.

The determination of paediatric reference intervals is both costly and time-consuming for most clinical laboratories due to the fact that blood samples from healthy children are not readily available, for ethical or practical reasons. In such circumstances, many laboratories adopt paediatric reference values from professional publications. One of them is a series of publications on the Canadian Laboratory Initiative on Paediatric Reference Intervals (CALIPER) (*3*-*5*). To date, the CALIPER database provides reference intervals for more than 100 laboratory parameters, and transference and verification studies on the applicability of the CALIPER database to major biochemical and immunoassay analysers have been published (*4*, *6*, *7*).

However, population-based reference values reflect the range of values in an examined reference population and may not necessarily be suitable for use in another population despite the same analytical method or analytical system. Therefore, according to the Clinical and Laboratory Standards Institute (CLSI) document, literature reference intervals must be verified before application on the local population (*8*-*10*). The available literature offers only studies on the transference of CALIPER reference values for different immunoassay analysers but not for transference and verification of CALIPER reference values on other world populations.

In the present study, verification of CALIPER derived reference values for ferritin and hormones on Beckman Coulter DxI600 immunoassay analyser was performed on a healthy population of the Croatian children and adolescents.

## Materials and methods

### Subjects

The study was conducted between July 2018 and April 2019 at Children’s Hospital Zagreb on a group of 295 healthy children and adolescents aged from 1 to 18 years. Subjects were selected using the direct *a posteriori* sampling method which involved the selection of outpatient healthy children and adolescents after collecting the blood samples (*8*). Hospital outpatients were recruited from general medical examination program, preoperative laboratory testing (*i.e.* tonsillectomy, orthopaedic and plastic surgery), orthopaedic examination program (*i.e.* flat foot, trigger finger, scoliosis) and ophthalmology patients *(i.e.* strabismus, astigmatism, cataract). Exclusion criteria included hospitalization, clinical history of acute or chronic diseases, metabolic disease, endocrine disease, routine haematological and biochemical laboratory test results outside the reference intervals for age. After the subjects were extracted from the hospital outpatients population by applying exclusion criteria they were randomly subdivided into age and gender subclasses.

According to current CLSI guidelines for verification of reference intervals using a small number of reference subjects, 20 samples were tested for each reference interval. The exception was the two gender-independent reference intervals spanning a wide age range; reference interval of ferritin for children aged 1 to 16 years and prolactin for children and adolescents aged 1 to 18 years. These reference intervals were examined in a group of 40 reference subjects (20 boys and 20 girls). This approach is own modification of standard verification procedure described in CLSI document (*8*). Besides, in our study, only the reference intervals for total testosterone for children older than 1.5 years were tested. Testosterone concentrations were higher in boys than girls during the first 1.5 years, after which time, testosterone concentration rapidly decreased in both gender (*4*). For total testosterone, both age and gender specific reference intervals for children until 1.5 years will be tested subsequently in next study.

Gender-independent reference intervals were investigated in the groups with equal number (10 boys and 10 girls) of children or adolescents of both sex.

The research has been complied with all the relevant national regulations and institutional policies and in accordance with the tenets of the Helsinki Declaration, and has been approved by the authors’ institutional ethical committee.

Fasting blood specimens were collected into gel serum separator tubes (Tube-Vacuette, Greiner Bio-One GmbH, Austria) in the morning hours from 8 to 10 am. Tubes were centrifuged and serum was separated within 1 hour after sampling, at room temperature. The residual serum after routine laboratory determinations was aliquoted within 4 hours after sampling and stored at - 20°C for maximum of 2 months. All samples were subjected to a single freeze-thaw cycle at time of analysis. Haemolysed, lipemic and icteric samples were not included in the study.

### Methods

Serum samples were analysed on the Beckman Coulter DxI600 immunoassay analyser (Beckman Coulter Inc, Brea, USA) by chemiluminescence immunoassay method (CLIA) for ferritin and 11 hormones: cortisol, dehydroepiandrosterone sulfate (DHEAS), follicle stimulating hormone (FSH), lutein stimulating hormone (LH), prolactin, progesterone, sex hormone binding globulin (SHBG), thyroid stimulating hormone (TSH), total testosterone, total thyroxine (T4) and total triiodothyronine (T3). Not all analytes were determined in all samples due to the limited serum volume. Analytical methods were used according to the manufacturer’s instructions for a reagents preparation, calibration procedures and analyser maintenance. The performance of methods was controlled using commercial lyophilised human serum samples at three concentration levels for each analyte as a part of internal quality control and by external quality controls as a part of external quality assessment scheme (Croatian Centre for Quality Assessment in Laboratory Medicine) (*11*).

If a sample concentration was less than the lower limit of detection for the immunoassay, the results were reported as less than value of the lower limit (*i.e.* LH and total testosterone).

The stability of the tested analytes were analysed in two serum samples. On the day of sampling (T0), the baseline values were determined, while stored values (T60) were analysed in frozen serum aliquots over a period of 2 months. Percentage deviation (the difference between the baseline concentration and its stored concentration converted into a % from the baseline concentration) of all tested analytes was compared with given quality specifications (*12*). If the percentage deviation is less than declared bias, the stability of analysed parameters not affected with a sample storage conditions.

Literature reference intervals for ferritin and hormones on a DxI600 immunoassay analyser were taken from CALIPER base (*3*, *4*).

### Statistical analysis

The distribution of the data was analysed using the D’Agostino-Pearson test. The homogeneity of the set of results was checked by Tukey test (*8*). Outliers were eliminated and replaced by new 24 reference subjects, so 20 results with no outliers were collected per each reference interval. Reference intervals were considered valid for transference if less than 3 of the 20 results were outside of the suggested reference interval. If 3 or 4 results were outside the limits, additional new 20 samples were collected, again free of outliers. A reference interval was verified if additional results met the specified criterion, no more than 2 of these new results were outside the CALIPER reference interval (*8*).

All statistical tests were performed using the statistical program MedCalc version 17.2. (Med Calc Statistical Software by Ostend, Belgium).

## Results

Serum samples from 139 male and 156 female children and adolescents were used to verify 51 reference intervals for ferritin and 11 hormones on the Beckman Coulter DxI600 immunoassay analyser according to CALIPER reference intervals (Table 1).

**Table 1:** CALIPER reference intervals verified on the Croatian children and adolescents

**Analyte, unit**	**Gender**	**Age** **(years)**	**CALIPER-** **reference** **intervals^(3, 4)^**	**Study results (number of outliers)**	**Study results (number of results outside CALIPER limits)**	**Study results (range)**
	M, F	1 - < 13	60.4 – 353.0	0	0	112.7 – 293.8
**Cortisol, nmol/L**	M, F	13 - < 16	83.6 – 472.0	0	0	132.7 – 438.4
	M, F	16 - < 18	104.0 – 535.0	1	0	280.5 – 476.3
	M, F	1 - < 6	< 1.01	3	0	0.05 – 0.60
	M, F	6 - < 9	0.13 – 2.55	0	1	0.10 – 2.60*
**DHEAS, μmol/L**	M, F	9 - < 13	0.43 – 5.62	0	0	0.60 – 5.10
	M	13 - < 16	1.79 – 11.29	0	1	1.60 – 11.10*
	M	16 - < 18	3.06 – 16.60	0	0	3.20 – 9.60
	F	13 - < 16	1.03 – 9.13	0	0	2.20 – 8.70
	F	16 - < 18	1.97 – 16.10	1	0	3.80 – 11.00
	M, F	1 - < 16	10.3 – 55.8	0	2	5.8 – 37.4*
**Ferritin, μg/L**	M	16 - < 18	18.7 – 102.0	0	0	25.6 – 88.0
	F	16 - < 18	3.2 – 75.1	0	0	4.8 – 42.5
	M	1 - < 9	0.23 – 2.32	1	0	0.34 – 1.37
	M	9 - < 12	0.56 – 4.98	1	1	0.53 – 2.20*
**FSH, IU/L**	M	12 - < 18	1.26 – 7.40	3	0	1.61 – 5.23
	F	1 - < 9	0.62 – 6.37	1	3 / 0	0.45 – 6.61^†^ / 1.02 – 5.16^‡^
	F	9 - < 12	0.91 – 7.83	1	0	0.99 – 5.04
	F	12 - < 18	0.59 – 10.20	0	0	1.54 – 9.23
	M, F	1 - < 5	< 2.14	1	0	< 0.20**^§^**
	M, F	5 - < 10	< 1.67	2	0	0.20 – 0.34
**LH, IU/L**	M	10 - < 14	< 3.28	0	0	0.20 – 3.12
	M	14 - < 18	0.81 – 8.96	0	0	1.26 – 6.20
	F	10 - < 14	< 8.09	3	0	0.20 – 6.70
	F	14 - < 18	1.59 – 19.00	0	0	1.89 – 14.88
	M, F	1 - < 9	< 2.73	0	1	0.49 – 2.79*
	M, F	9 - < 13	< 4.48	0	0	0.53 – 4.08
**Progesterone, nmol/L**	M	13 - < 18	0.60 – 5.22	0	0	0.85 – 4.68
	F	13 - < 18	0.80 – 38.5	1	0	1.04 – 4.66
**Prolactin, μmol/L**	M, F	1 - < 18	3.21 – 18.50	2	1	2.81 – 13.92*
**SHBG, nmol/L**	M, F	1 - < 8	53.2 – 174.0	0	2	59.9 – 182.6*
	M, F	8 - < 11	45.4 – 144.0	0	3 / 0	32.0 – 177.9^†^ / 46.3 – 110.1^‡^
	M, F	11 - < 13	15.9 – 132.0	1	0	18.8 – 97.1
	M	13 - < 18	10.5 – 75.3	0	0	12.5 – 49.4
	F	13 - < 18	18.1 – 95.8	0	0	21.8 – 85.4
	M, F	1.5 - < 7	< 0.35^§^	0	0	< 0.35^§^
	M, F	7 - < 9	< 0.62	0	0	< 0.35^§^
**Analyte, unit**	**Gender**	**Age** **(years)**	**CALIPER-** **reference** **intervals^(3, 4)^**	**Study results (number of outliers)**	**Study results (number of results outside CALIPER limits)**	**Study results (range)**
**Total testosterone, nmol/L**	M, F	9 - < 12	< 1.63	0	0	0.35 – 0.87
	M	12 - < 18	0.38 – 19.60	0	0	0.73 – 17.48
	F	12 - < 15	< 2.26	0	0	0.35 – 2.25
	F	15 - < 18	0.62 – 2.98	0	1	0.95 – 3.37*
	M, F	1 - < 12	1.84 – 3.12	0	1	1.80 – 2.70*
**T3, nmol/L**	M	12 - < 16	1.77 – 3.16	0	0	1.80 – 2.70
	F	12 - < 16	1.54 – 3.00	0	0	1.60 – 2.40
	M, F	16 - < 18	1.51 – 2.95	1	0	1.70 – 2.30
	M, F	1 - < 4	76 – 168	0	0	93 – 144
**T4, nmol/L**	M, F	4 - < 14	70 – 130	0	0	83 – 130
	M	14 - < 18	68 – 119	0	0	73 – 119
	F	14 - < 18	75 – 141	0	0	84 - 136
**TSH, mIU/L**	M, F	1 - < 12	0.79 – 5.85	1	0	1.33 – 4.01
	M, F	12 - < 18	0.68 – 3.35	0	0	1.07 – 3.21
M - male. F - female. *The results measured in our study with less than 3 out of 20 samples outside the CALIPER reference interval. ^†^the results measured in our study with 3 out of 20 samples outside the CALIPER reference interval. ^‡^the results measured in our study in the additional group of reference subjects. ^§^results below the lower limit of detection (LLD) of the immunoassay denoted with „< LLD“. DHEAS - dehydroepiandrosterone sulfate. FSH - follicle stimulating hormone. LH - lutein stimulating hormone. SHBG - sex hormone binding globulin. TSH - thyroid stimulating hormone. T4 - total thyroxine. T3 - total triiodothyronine.						

For the 40 reference intervals, the results of all 20 samples were within the specified interval limits, whereas for 9 reference intervals, ≤ 2 of the 20 samples were outside the reported limits. The latter included one reference interval of ferritin, FSH, progesterone, prolactin, SHBG, total testosterone, TT3 and two reference intervals for DHEAS. After first measurements, two reference intervals were invalid for transference; reference intervals of FSH for female aged 1 to 9 years and SHBG for children aged 8 to 11 years. Two out of 20 reference values had FSH concentrations above the upper reference limit of 6.37 UI/L and one values was below the lower reference limit of 0.62 IU/L. The SHBG serum concentration was below the lower limit of 45.4 nmol/L in 2 reference samples and one samples had a value above the upper reference limit of 144.0 nmol/L. The all results of 20 another reference subjects per specific age and gender subclasses for FSH and SHBG were within the specified CALIPER interval limits (Table 1).

Comparison of the preanalytical features of the CALIPER study and our study is presented in Table 2.

**Table 2:** Comparison of preanalytical factors

**Preanalytical factor**	**CALIPER study^(3,4)^**	**Test study**
Reference population	Canadian	Croatian
Subject type	healthy community	hospital outpatients
Sampling approach	direct	direct (*a posteriori*)
Time of blood collection	all day	from 8 to 10 am
Blood sampling tube	serum separator tube (SST Becton Dickinson)	serum separator tube (Vacuette, Greiner Bio-One)
Separation and aliquoting of serum	within 4 h	within 4 h
Serum storage	- 80°C	- 20°C (up to 2 months)

## Discussion

The results of our study showed that CALIPER reference values for ferritin, cortisol, DHEAS, FSH, LH, prolactin, progesterone, SHBG, TSH, total testosterone, T4 and T3 can be implemented into our laboratory and clinical practice. However, the Children’s Hospital Zagreb provides the highest level of health care for children and adolescents from all over Croatia, so our results can be applied to all Croatian laboratories that determine these analytes on a Beckman Coulter DxI600 analyser.

The results of the laboratory test are widely used in medical decision making, from diagnosis to monitoring of the effectiveness of therapy. The interpretation of laboratory reports is most often based on a comparison of test results with reference intervals. Paediatric reference values are a special category of reference intervals covering a period of intense physiological growth and development, so multiple subclasses of reference intervals by age and gender are differentiated. The establishment of paediatric reference intervals is a challenging process because of ethical standards, financial constraints and time frames. For this reason, reference intervals declared by the manufacturer or the ones taken from the professional literature are very common sources of reference intervals in routine medical laboratory. According to the CLSI guideline, the transfer of reference intervals from the original reference values study or other sources such as professional recommendations or manufacturer’s package inserts to the local population requires the comparability of methodology and preanalytical processes between original and local studies (*8*).

This study presents verification of reference intervals taken from the CALIPER analyser-specific database (*4*). The study included children and adolescents aged 1 to 18 years. Since intense growth and sexual maturation during this period in life is influenced by significant changes in the concentration of ferritin and hormones, 51 reference intervals for adrenal, fertility and thyroid hormones, besides ferritin were analysed in this study.

The concentration of all analytes in this study was determined on Beckman Coulter DxI600 immunoassay analyser using the manufacturer’s reagents and calibrators. The assay performance characteristics in our laboratory conditions met the manufacturer’s and laboratory quality specifications for imprecision and bias. Thus, there is an analytical equivalence between method/analyser used in the CALIPER study and our local laboratory settings (*3*, *4*).

However, there are certain differences in preanalytical procedures between the two studies. While the CALIPER study was conducted on Canadian healthy community children and adolescents aged 0 to 19 years, this study included Croatian healthy hospital outpatient children and adolescents aged 1 to 18 years. As health does not have a universal definition, the selection of these study reference subjects was based on the exclusion criteria for potentially unhealthy children and adolescents. The selected subjects from the hospital outpatient populations did not have other conditions that could affect tested results. Thus, there is an acceptable group of reference subjects.

Another difference is the sampling approach between the CALIPER and this study. The exclusion criteria was applied before blood collection performed in the CALIPER study, while subjects in this study were selected after the blood sampling. However, both of these sampling approach belong to recommended direct sampling methods in which subject were selected from a reference population using well defined criteria (*1*, *8*).

Many other preanalytical factors may affect the concentration of biomarkers in blood. The biological preanalytical variables include physiologic factors such as fasting or non-fasting condition and circadian rhythm of hormone production while the preanalytical methodological factors involve sample collection and processing (*8*). The standardization of patients preparation before blood collection reduce sources of variability test results. Therefore, all children and adolescents in this study were tested under routine standardized conditions for fasting status before collecting blood between 8 and 10 am according the EFLM strategy for harmonization of the preanalytical phase (*13*). Unlike this study, in the CALIPER study blood samples were collected throughout all day, fasting status was not been required, but time of blood collection and time since last meal were recorded (*3*).

Also, there are differences in type of blood sampling tubes and serum storage conditions. The possible effect of the type of blood sampling tube on the analyte concentration has been described in the literature, but has not been investigated in this study (*14*). On the other hand, the serum stability during the storage period at - 20°C was examined since the stability of the frozen sample was not defined by manufacturer. The concentration of all tested analytes was stable over period of 2 months of storage at - 20°C because the percentage deviations of all tested parameters are less than declared biases (data not shown) (*12*).

Verification of the CALIPER reference intervals was performed by evaluating reference interval using 20 reference samples. After the first set of measurements all results were within the limits of the reference interval in 40 of the 51 (78%) reference intervals. One to two measured results outside the limits of the reference interval were observed in 9 of 51 (18%) reference intervals for 8 different analytes. This means that the transfer of reference intervals from CALIPER database was possible after the first set of measurements for almost 96% of the tested reference intervals. The collection of additional set of samples was only required for two reference intervals, FSH for females aged 1 to 9 years and SHBG for children aged 8 to 11 years. All three results outside the limits of the above mentioned FSH and SHBG reference intervals were equally distributed below the lower and upper limits of the reference intervals. All results in the additional groups of 20 reference subjects for specific age groups met the required criteria for transference of CALIPER reference intervals for FSH and SHBG on the Beckman Coulter DxI600 immunoassay analyser.

The limitation of study is the inability to compare these results with other population-based verification studies of the CALIPER reference values. Although the CALIPER program is committed to the global harmonization of paediatric reference intervals, only the results of determining reference intervals using new analytical technologies are reported in the literature (*3*). Furthermore, the editors of some professional journals believe that publishing the CALIPER verification studies would overload the journal space. Although we understand editorial policy, we believe that global harmonization of paediatric reference values require population studies to examine the effect of regional preanalytical factors.

In conclusion, use of harmonized reference values would be reduced unnecessary repetition of laboratory testing, improved the clinical interpretation of laboratory test results and enabled better comparison of literature data.
